# Strain Engineering in Ni-Co-Mn-Sn Magnetic Shape Memory Alloys: Influence on the Magnetic Properties and Martensitic Transformation

**DOI:** 10.3390/ma15175889

**Published:** 2022-08-26

**Authors:** Qinhan Xia, Changlong Tan, Binglun Han, Xiaohua Tian, Lei Zhao, Wenbin Zhao, Tianyou Ma, Cheng Wang, Kun Zhang

**Affiliations:** 1 School of Science, Harbin University of Science and Technology, Harbin 150080, China; 2 School of Materials Science and Chemical Engineering, Harbin University of Science and Technology, Harbin 150080, China; 3 School of Electrical and Electronic Engineering, Harbin University of Science and Technology, Harbin 150080, China; 4 Shenyang National Laboratory for Materials Science, Institute of Metal Research, Chinese Academy of Sciences, Shenyang 110016, China

**Keywords:** martensitic transformation, ferromagnetic shape memory alloys, first-principal calculations, Ni-Co-Mn-Sn, strain engineering

## Abstract

Ni-Mn-Sn ferromagnetic shape memory alloys, which can be stimulated by an external magnetic field, exhibit a fast response and have aroused wide attention. However, the fixed and restricted working temperature range has become a challenge in practical application. Here, we introduced strain engineering, which is an effective strategy to dynamically tune the broad working temperature region of Ni-Co-Mn-Sn alloys. The influence of biaxial strain on the working temperature range of Ni-Co-Mn-Sn alloy was systematically investigated by the ab initio calculation. These calculation results show a wide working temperature range (200 K) in Ni_14_Co_2_Mn_13_Sn_3_ FSMAs can be achieved with a slight strain from 1.5% to −1.5%, and this wide working temperature range makes Ni_14_Co_2_Mn_13_Sn_3_ meet the application requirements for both low-temperature and high-temperature (151–356 K) simultaneously. Moreover, strain engineering is demonstrated to be an effective method of tuning martensitic transformation. The strain can enhance the stability of the Ni_14_Co_2_Mn_13_Sn_3_ martensitic phase. In addition, the effects of strain on the magnetic properties and the martensitic transformation are explained by the electronic structure in Ni_14_Co_2_Mn_13_Sn_3_ FSMAs.

## 1. Introduction

Ni-Mn-Sn ferromagnetic shape memory alloys (FSMAs) with various related magnetic effects, including excellent magnetocaloric effect (MCE) [1,2], extraordinary magnetic shape memory effect (MSME) [3,4,5], and magnetoresistance effect (MR) [6,7]. These multifunctional properties are attributable to the coupling between the magnetic and structural transitions by the magnetic field [8,9,10], i.e., magnetic field-induced martensitic transformation (MFIMT). It is the magnetic field-driven shape memory behavior that makes ferromagnetic shape memory alloys different from conventional shape memory alloys, which must be actuated through temperature. The application of magnetic fields is fast and easy, and its fast response to magnetic fields makes this alloy more widely used than conventional shape memory alloys. Moreover, Ni-Mn-Sn FSMAs are cheap, non-toxic, and have simple fabrication processes, which highlights their advantages over conventional shape memory alloys. Although Ni-Mn-Sn FAMAs have so many excellent properties, the relatively narrow working temperature range is still a key drawback in extensive practical application [11,12]. Previous studies have pointed out that the narrow working temperature of Ni-Mn-Sn alloys is mainly near room temperature or slightly below it [13,14,15,16,17,18,19,20], which is just more beneficial to magnetic solid-state refrigeration. However, for the automotive, manufacturing, and energy exploration industries, the high working temperature region of the alloys is needed [21,22]. Similarly, a working temperature range lower than 270 K is needed for several space solutions [23]. In addition, the working temperature is very sensitive to constituent elements. That is, the working temperature region with fixed components is also fixed, which also increases the difficulty of widening the working temperature region. Therefore, obtaining the adjustable working temperature range is an urgent problem to be solved in Ni-Mn-Sn alloys.

Strain engineering is an efficient method to enhance the properties of functional materials [24,25,26,27]. Experimentally, Huang et al. found that uniaxial strain will be an effective means to control thermal effects (such as giant MCE and elastocaloric effects). A significant cooling level of about 4 K is measured when the strain is released [28]. Yang et al. observed that the martensitic transformation temperature (*T*_M_) increased with the increase in uniaxial strain in Ni_43.5_Co_6.5_Mn_39_Sn_11_ [29]. In addition, Zhao et al. also measured that the refrigerating temperature region increased by 6 K to 10 K in Ni_43_Co_7_Mn_39_Sn_11_ films with applying strain [30]. It can be seen that stress engineering is a very effective method to improve many properties of the system. However, few studies have been able to draw on any systematic research on the influence of biaxial strain on the working temperature in the Ni-Mn-Sn system.

The stable MFIMT and the dynamical working temperature are necessary for a tunable broad working temperature region in Ni-Mn-Sn. For a stable MFIMT, the alloys must be ferromagnetic in the austenitic phase (FM) and antiferromagnetic in the martensitic phase (AF) for a stable MFIMT (AFM). For the dynamical working temperature, the alloys need to require two conditions. (1) Both T_M_ and Curie temperature (*T*_C_) need to be dynamic. (2) Keeping *T*_M_ lower than *T*_C_. In addition, from the view of calculations, the large magnetization (Δ*M*) between the austenitic and martensitic phases is beneficial to the stable MFIMT [5]. The total energy difference (Δ*E*_A-M_) between the austenitic phase and martensitic phase and the total energy difference (Δ*E*_P-F_) between the ferromagnetic austenitic phase and paramagnetic austenitic phase play important roles in predicting the working temperature in alloys [5,31,32]. The *T*_M_ and *T*_C_ are increasing with the increase in the Δ*E*_A-M_ and Δ*E*_P-F_, respectively [31,33]. In light of this, we calculated the Δ*M*, Δ*E*_A-M*,*_ and Δ*E*_P-F_ with the different strains through first-principles calculations. The results show that the value of Δ*M* of Ni-Mn-Sn alloys is too small to meet the stable MFIMT. Thus, to improve the value of Δ*M* and Δ*E*_P-F_, we choose the method of doping elements (Co to substitute for Ni atoms) [34,35]. In this way, Ni-Co-Mn-Sn alloys not only have a stable MFIMT but also have a dynamic working temperature. It is a high-quality candidate material for dynamically adjusting the working temperature region.

In the present paper, we aim to propose a strategy to adjust the broad working temperature of Ni-Co-Mn-Sn alloys by strain (from 200 K to 370 K). By using the first-principles calculation, the influences of strain on the magnetic properties, the martensitic phase transformation (MPT), and the working temperature of the alloys have been comprehensively studied. According to the results, a small strain can significantly change the working temperature and maintain the stable MFIMT, and the wide working temperature region of 168 K to 330 K can be predicted under strain from 0% to −1.5% in Ni_14_Co_2_Mn_13_Sn_3_ alloys. In addition, we discussed the physical mechanism of magnetic and martensitic transformation of the Ni_14_Co_2_Mn_13_Sn_3_ alloy through the electronic structure. 

## 2. Calculation Method

**Figure 1:**
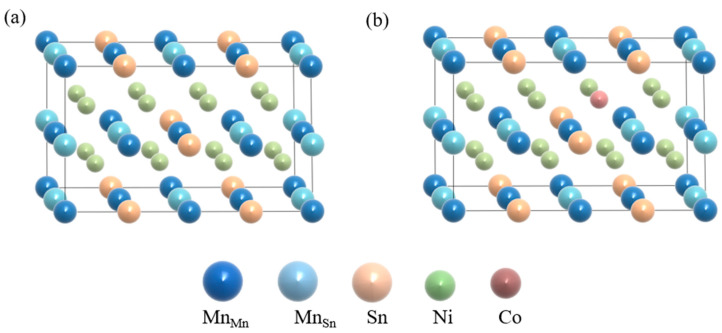
(**a**) Crystallographic structure in Ni_16_Mn_13_Sn_3_ austenitic phase. (**b**) Crystallographic structure in Ni_15_CoMn_13_Sn_3_ austenitic phase.

The Vienna Ab initio Simulation Package (VASP) code is used to reveal the magnetic properties [36,37], phase structures, and electronic structures of Ni-Co-Mn-Sn FSMAs. All works were performed on the basis of the density functional theory (DFT). As the exchange–correlation functional, we used the Perdew–Burke–Ernzerhof (PBE) method and the generalized gradient approximation (GGA) [38]. For Ni-Mn-Sn FSMAs, a k-mesh of 3 × 6 × 6 is used for two phases. The cut-off energy is 500 eV. The *L*2_1_ austenite structure ($Fm\bar{3}m$) of Ni_16_Mn_13_Sn_3_ with three inequivalent Wyckoff positions (4a, 4b, 8c) is shown in Figure 1a. The Sb and Mn atoms occupy 4a (0, 0, 0) and 4b (0.50, 0.50, 0.50) positions respectively and Ni atoms occupy the 8c ((0.25, 0.25, 0.25) and (0.75, 0.75, 0.75)) sites. In addition, the calculation method used in the transformation process from austenite to martensite is tetragonal distortion. That is, on the premise of keeping the cell volume unchanged, the optimized austenite structure is subjected to lattice distortion with different tetragonal distortion rates c/a so as to obtain the most stable martensite structure. As seen in Figure 1b, we substituted Co atoms for Ni atoms directly in our study, and Mn_Sn_ is the designation for the excess Mn atoms at the deficient Sn atoms. The Mn atoms that remain at their sites are called Mn_Mn_ in the Ni_16−*x*_Co*_x_*Mn_13_Sn_3_ (*x* = 0, 1, 2) FSMAs. For both the austenitic and martensitic phases, we calculated two situations: the magnetic properties of Ni_16−*x*_Co*_x_*Mn_13_Sn_3_ (*x* = 0, 1, 2) alloys are FM states and AFM states. The FM configuration was set by magnetic moments of all Mn atoms (Mn_Sn_ and Mn_Mn_) parallel to each other. AFM configuration was decided by magnetic moments of Mn_Sn_, which are opposite in the direction of the magnetic moments of the Mn_Mn_. According to the VASP user manual [39], the calculation of spin polarization requires the parameter ISPIN = 2, while the setting of FM and AFM is determined by the parameter MAGMOM. Therefore, the spin polarization of both ferromagnets and antiferromagnets can be achieved by VASP. In first-principles calculations, we simulate biaxial strain by changing lattice vectors directly. That is, fixing the lattice constant in the c-axis while relaxing the lattice constants in the a-axis and b-axis. It is worth mentioning that 0% represents no deformation, positive deformation represents stretching, and negative deformation represents compression.

## 3. Results and Discussions

**Figure 2:**
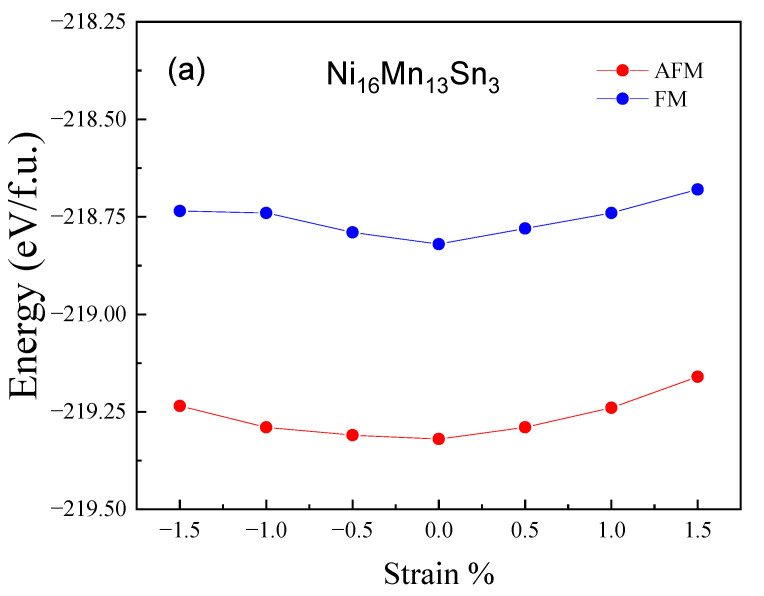

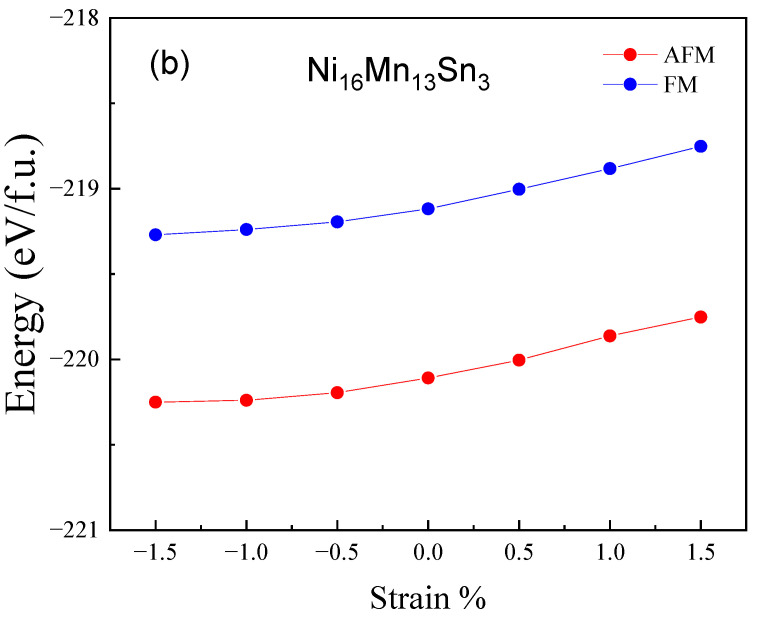
Strain change (−1.5~1.5%) of the total energies of Ni_16_Mn_13_Sn_3_ alloys. (**a**) the austenitic phase. (**b**) the martensitic phase.

**Figure 3:**
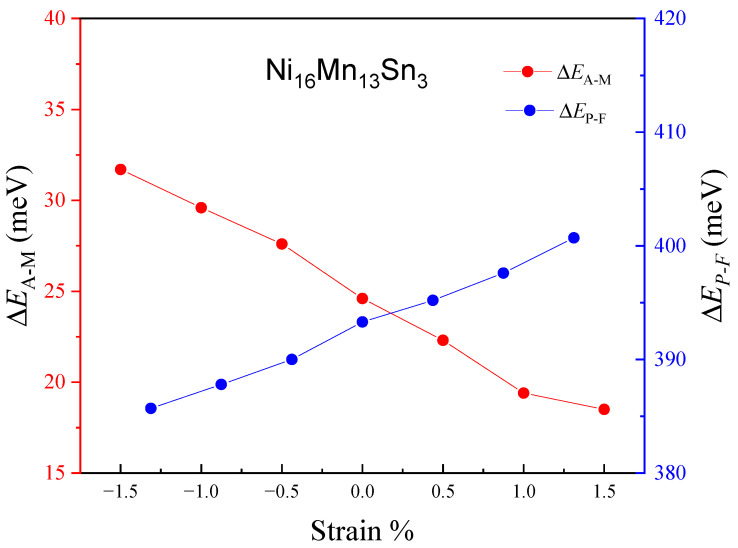
Strain change (−1.5–1.5%) of Δ*E*_A-M_ and Δ*E*_P-F_ for Ni_16_Mn_13_Sn_3_.

First, we investigated the two phasic structures, martensitic transition and magnetic properties of the Ni_16_Mn_13_Sn_3_. Table 1 shows the results of our calculations for the magnetic properties and equilibrium lattice parameters of the Ni_16_Mn_13_Sn_3_ alloys. For the Ni_16_Mn_13_Sn_3_ austenitic phase, the AFM state of the alloy is low energy, and the equilibrium lattice parameter is 5.94 Å. The magnetic ground state and lattice constant are in good agreement with other theoretical values [40]. In the Ni_16_Mn_13_Sn_3_ martensitic phase, the FM state has higher energy and is more unstable than the AFM state at *c/a*~1.35. That is, austenite and martensite phase are AFM states under 0% strain. This is also consistent with the theoretical results [3]. According to the energy corresponding to 0% strain in Figure 2, the energy of AFM austenite is higher than that of AFM martensite, so MPT will occur, which is a prerequisite for shape memory alloys. This is also verified experimentally [41]. The above results confirm the correctness of our calculation, so we can apply biaxial strain based on it, and then we calculated the total energies *E* of the Ni_16_Mn_13_Sn_3_ austenitic phase and martensitic phase with strain (−1.5~1.5%), as shown in Figure 2a,b respectively, to reveal the effect of strain on the phase structures, MPT, and magnetic properties. Figure 2a indicates that the energy of the AFM state is lower than that in the FM state for the austenite phase, and the total energies *E* of Ni_16_Mn_13_Sn_3_ austenitic phase firstly decrease with strain from 1.5% to 0% and then increase with strain from 0% to −1.5%. For the Ni_16_Mn_13_Sn_3_ martensitic phase, the total energy *E* of both FM and AFM increases with strain from 1.5% to −1.5%, and the AFM state energies are lower than the FM state energies. Therefore, we can conclude that austenite and martensite of Ni_16_Mn_13_Sn_3_ alloy are still in AFM state under the application of biaxial strain; that is, the biaxial strain will not affect the magnetic ground state of the system. In addition, the value of Δ*E*_A-M_ and Δ*E*_P-F_ in Ni_16_Mn_13_Sn_3_ alloys under strain (−1.5~1.5%) are shown in Figure 3 to show the impact of strain on *T*_M_ and *T*_C_. It is obvious that the Δ*E*_A-M_ increases with strain, while the Δ*E*_P-F_ decrease with strain under strain from 1.5% to −1.5%. This shows that *T*_M_ and *T*_C_ increase with the increase in Δ*E*_A-M_ and Δ*E*_P-F_, respectively. According to the results, applying strain can tune the working temperature of Ni_16_Mn_13_Sn_3_ alloys. Based on the above results, it can be concluded that the stability of austenite will be reduced no matter whether biaxial compressive strain or biaxial tensile strain is applied. However, the stability of martensite increases with compressive strain and decreases with tensile strain. Moreover, the biaxial strain does not affect the occurrence of MPT and the most stable magnetic configuration of each phase.

In order to tune the working temperature of alloys, alloys must have stable MFIMT. The Δ*M* is larger, and the MFIMT is more stable [41,42]. Therefore, we calculated the Δ*M* of Ni_16_Mn_13_Sn_3_ alloys with strain in Table 1. Table 1 further accurately shows that the Δ*M* of Ni_16_Mn_13_Sn_3_ is very small, and the strain has a weak effect on the value of Δ*M* (0.06–0.01 μB). The low Δ*M* cannot satisfy the stable MFIMT. Therefore, the biaxial strain alone cannot satisfy the stable MFIMT, which is a necessary condition for an adjusted wide working temperature region. Fortunately, the Co element enhances ferromagnetism in the austenitic phase and *T*_C_ of Ni_16_Mn_13_Sn_3_ alloys. Thus, the strain may be an efficacious strategy to adjust the wide working temperature of Ni-Co-Mn-Sn.

The impact of Co doping on the physical properties of the Ni-Co-Mn-Sn system, particularly on the operating temperature, must also be taken into account. We first evaluate the equilibrium lattice parameters and magnetic properties of the Ni_16−*x*_Co*_x_*Mn_13_Sn_3_ (*x* = 1, 2) and present them in Table 1 to show the change in phase structures, MPT, and magnetic properties of the Ni_16_Mn_13_Sn_3_ with Co doping. The findings demonstrate that as Co increases, the equilibrium lattice parameters of the Ni_16−*x*_Co*_x_*Mn_13_Sn_3_ (*x* = 1, 2) austenitic phases increase from 5.91 Å to 5.93 Å. The change of Ni_16−*x*_Co*_x_*Mn_13_Sn_3_ (*x* = 1, 2) lattice constant can be attributed to the fact that the atomic radius of the substitution elements is slightly larger than that of the substituted element, and the lattice parameters are close to the value of the experiment (5.987 Å) and theory (5.973 Å) [43,44]. The origin of the experimental error is the slight difference between the actual compound and the nominal compound, and then the DFT calculation is carried out at T = 0 K, while the equilibrium lattice constant measured by XRD is carried out at room temperature. The theoretical error may be caused by the error between different calculation software. The austenitic Ni_14_Co_2_Mn_13_Sn_3_ phase is the FM state, whereas the martensitic phase is the AFM state, and there is a large Δ*M* (5.48 μB) between these two phases as shown in Table 1. This is also consistent with the experimental facts (6.68 μB) [45]. In short, the Ni_14_Co_2_Mn_13_Sn_3_ alloys meet the stable MFIMT, and the strain method may be an efficient strategy to tune the wide working temperature of Ni_14_Co_2_Mn_13_Sn_3_ alloys.

**Figure 4:**
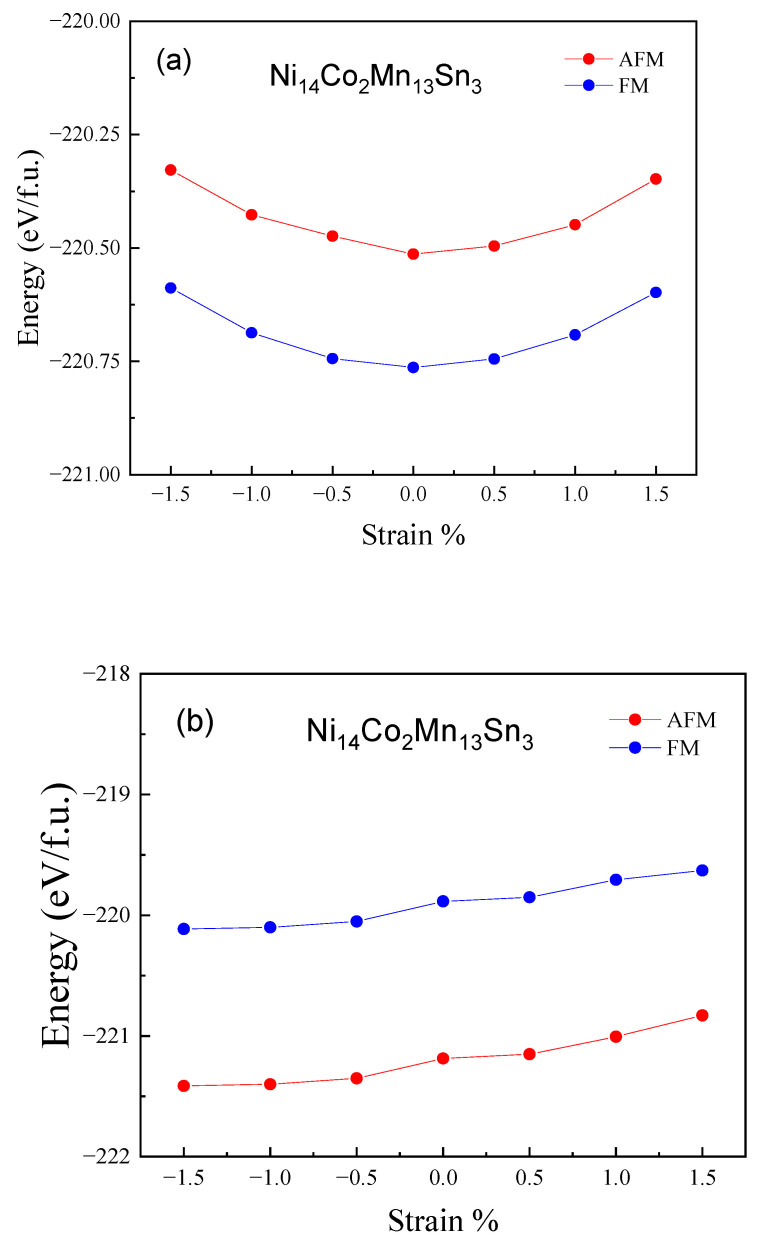
Strain change (−1.5~1.5%) of the total energies of Ni_14_Co_2_Mn_13_Sn_3_ alloys. (**a**) The austenitic phase. (**b**) the martensitic phase.

**Figure 5:**
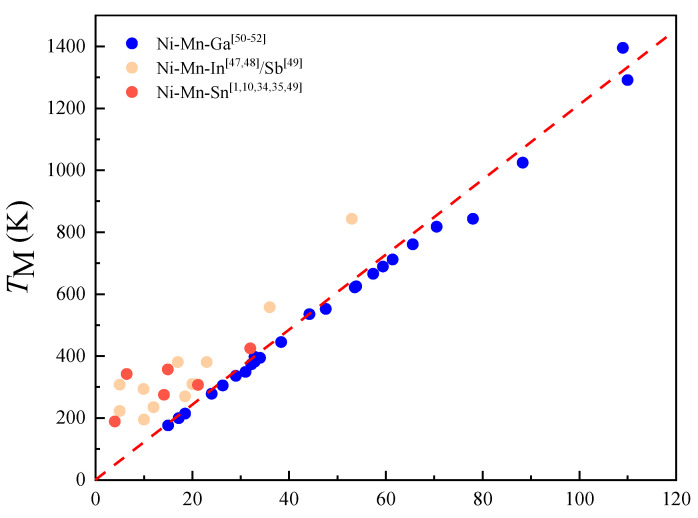
Relationship of Δ*E*_A-M_ and experimental *T*_M_ of alloys.

**Figure 6:**
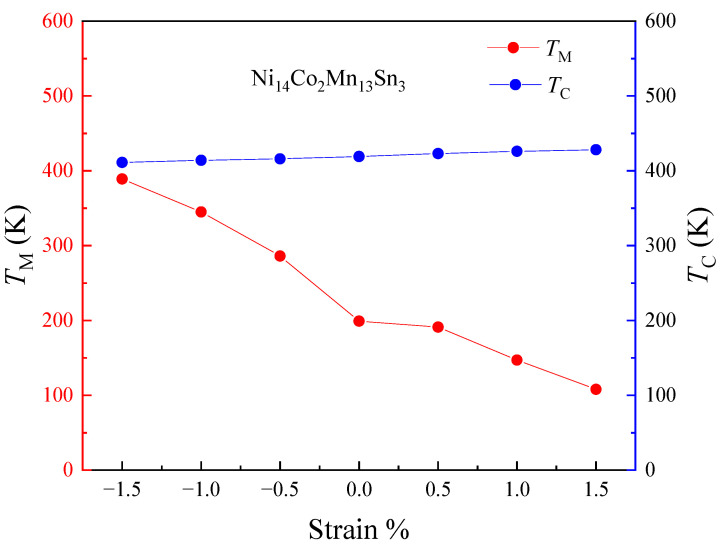
Strain change (−1.5~1.5%) of the *T*_M_ and the *T*_C_ in Ni_14_Co_2_Mn_13_Sn_3_.

Subsequently, we calculated the total energies of Ni_14_Co_2_Mn_13_Sn_3_ alloys with strain (−1.5~1.5%) and shown in Figure 4 to show the impact of the magnetic properties and working temperature on this alloy. It is obvious that the total energies of austenitic phases firstly decrease with strain (−1.5~0%) and then increase with strain (0–1.5%), and FM states are the most stable magnetic configuration for austenitic phases. The total energies of martensitic phases increase with strain (−1.5~1.5%), as shown in Figure 4b. Moreover, the most stable magnetic configuration of martensitic phases is AFM states. Combined with (a) and (b) of Figure 4, it can be seen that the energy of AFM martensite is always less than that of FM austenite under the action of biaxial strain. It shows that the alloy will undergo magnetic structure coupling transformation, which further confirms that there is a large magnetic moment difference in the system. To show the change of *T*_M_ and *T*_C_ of Ni_14_Co_2_Mn_13_Sn_3_ alloys with strain, we listed the Δ*E*_A-M_ and Δ*E*_P-F_ in Table 2. The value of Δ*E*_A-M_ increase with strain from 1.5% to −1.5%, while the value of Δ*E*_P-F_ decrease with strain from 1.5% to −1.5%. The results show the strain can increase *T*_M_ and decrease *T*_C_. In consideration of the wide working temperature of Ni_14_Co_2_Mn_13_Sn_3_ alloys, one of the conditions is that *T*_C_ must be higher than *T*_M_. Therefore, we need to evaluate the value of *T*_M_ and *T*_C_. Generally, the *T*_M_ and *T*_C_ increase linearly with Δ*E*_A-M_ and Δ*E*_P-F_ in Ni-Mn-based Heusler alloys. To further explore the relationship between *T*_M_ and Δ*E*_A-M_, as depicted in Figure 5, we made the *T*_M_ and Δ*E*_A-M_ fitting curves [1,10,34,35,46,47,48,49,50,51]. According to the Heisenberg model and Stoner theory [52], the relationship of *T*_M_ and Δ*E*_A-M_ is represented by Δ*E*_P-F =_ −k_B_*T*c*M/M_0_*, where *M* is the magnetic moment at *T* ≠ 0 K, and *M*_0_ is the equilibrium magnetic moment at *T* = 0 K [53]. Based on it, we calculated the *T*_M_ and *T*_C_ of Ni_14_Co_2_Mn_13_Sn_3_ alloys with strain in Figure 6. It shows that the *T*_M_ and *T*_C_ increase with strain, and the *T*_M_ is lower than *T*_C_ with strain (−1.5~1.5%), which indicates that the alloy has been fully qualified to dynamically adjust the wide working temperature range. In addition, the changing trend of *T*_M_ is consistent with the experiment in other Ni-Mn based [54]; that is, the working temperature moves to a high temperature under the compressive strain. Then, with strain from 0% to −1.5%, the Ni_14_Co_2_Mn_13_Sn_3_ FSMAs show a tunable wide working temperature (from 168 K to 330 K), which meets the application in different temperatures. The operating temperature range of Ni_14_Co_2_Mn_13_Sn_3_ is 160 K; the wide range may be overestimated compared to experimental values. In short, the strain method is an effective way to tune effectively by using the strain approach for Ni_14_Co_2_Mn_13_Sn_3_ alloys.

**Figure 7:**
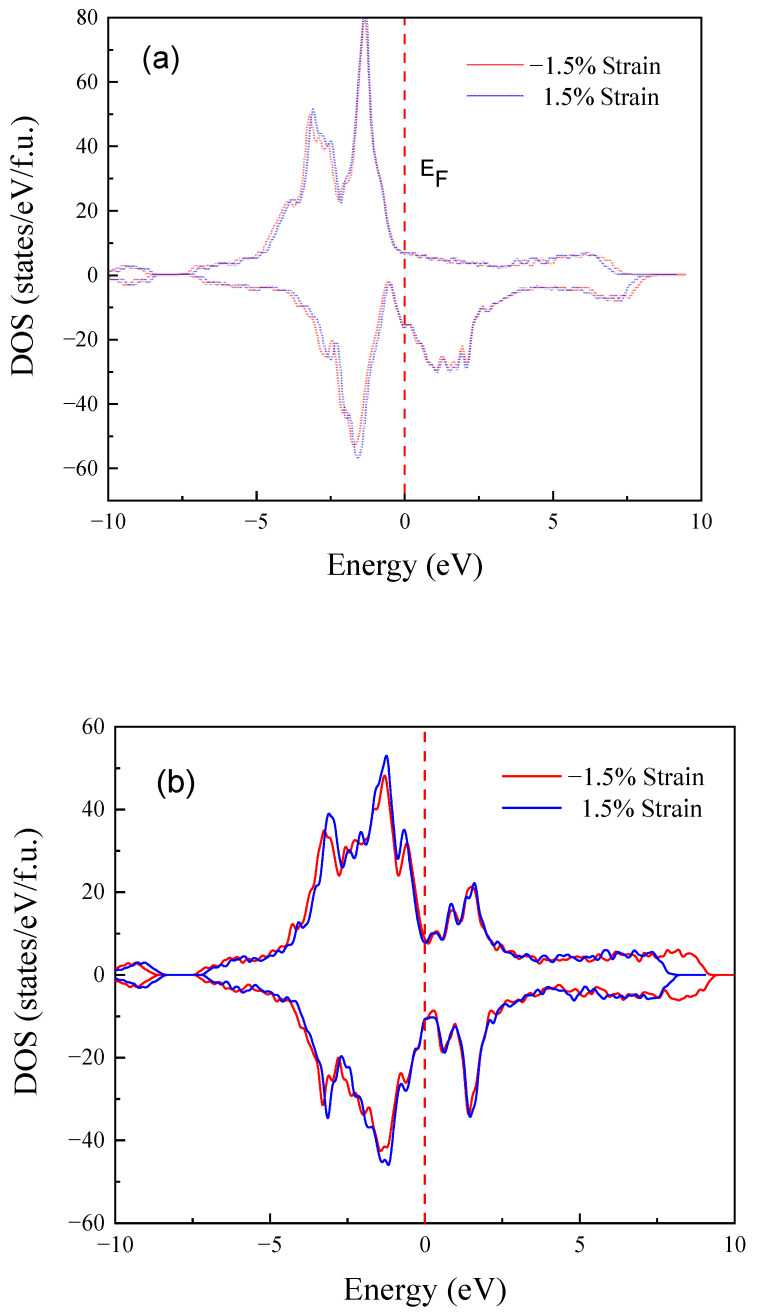
Strain change (−1.5%, 1.5%) of the TDOS of Ni_14_Co_2_Mn_13_Sn_3_ alloys. (**a**) the austenitic phase. (**b**) the martensitic phase.

**Figure 8:**
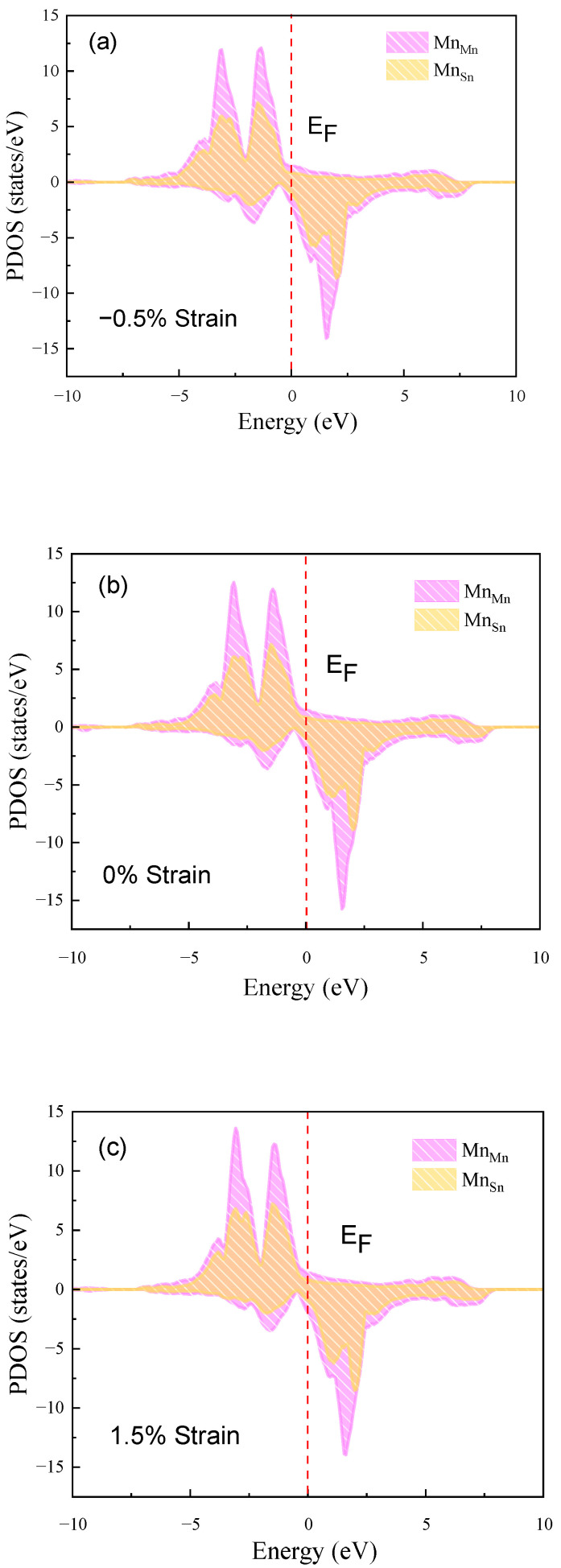
Strain change (−1.5%, 0%, 1.5%) of the PDOS of Ni_14_Co_2_Mn_13_Sn_3_ austenitic phase. (**a**) −1.5% strain, (**b**) 0% strain, (**c**) 1.5% strain.

**Figure 9:**
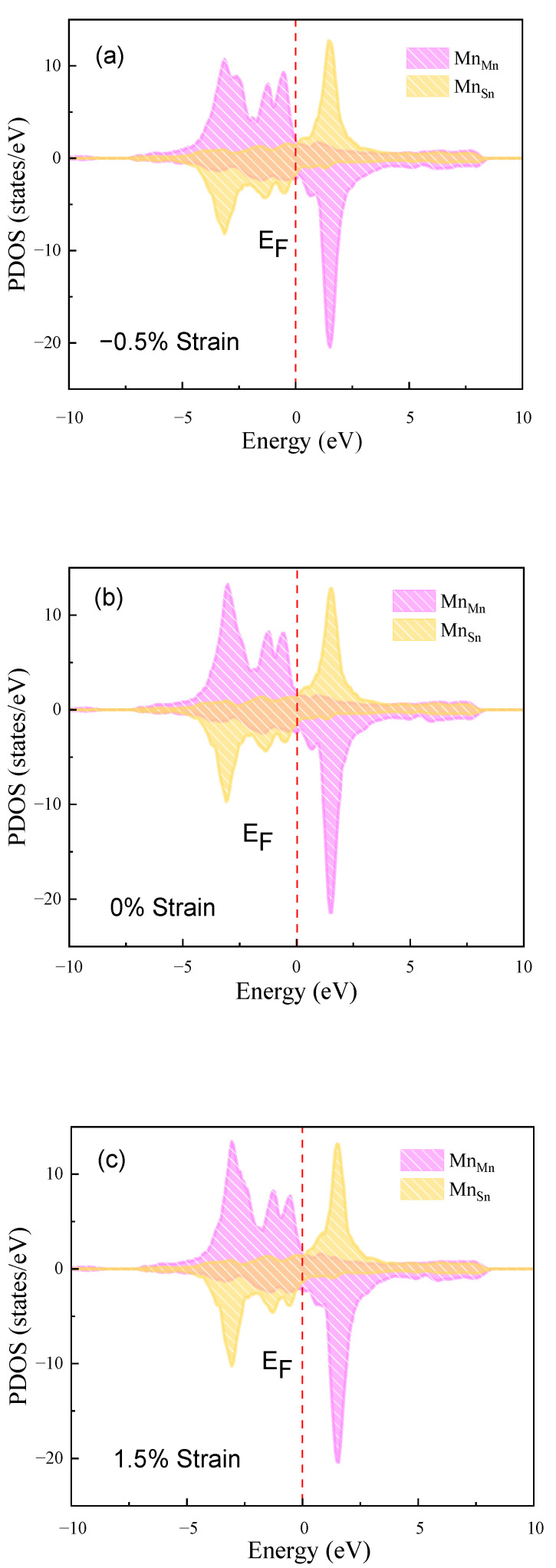
Strain change (−1.5%, 0%, 1.5%) of the PDOS of Ni_14_Co_2_Mn_13_Sn_3_ martensitic phase. (**a**) −1.5% strain, (**b**) 0% strain, (**c**) 1.5% strain.

The total density of states (TDOS) of austenite and martensite in Ni_14_Co_2_Mn_13_Sn_3_ and the partial density of states (PDOS) of Mn_Mn_ and Mn_Sn_ of austenite and martensite in Ni_14_Co_2_Mn_13_Sn_3_ are shown in Figure 7, Figure 8 and Figure 9 respectively to further illuminate the physical mechanism of the MPT and magnetic properties [55,56]. In addition, the phase stability is strongly dependent upon the TDOS at the Fermi level plays (E_F_) [57,58]. Usually, the low TDOS indicates the stable phase in Ni_14_Co_2_Mn_13_Sn_3_ FSMAs. Figure 7a shows that the TDOS at E_F_ of strain (−1.5%, 1.5%) is similar in Ni_14_Co_2_Mn_13_Sn_3_ austenitic phases. The strain has a weak effect on austenitic phase stability. Moreover, for martensitic phases, the TDOS of −1.5% strain is lower than the TDOS of 1.5% strain at E_F_, as shown in Figure 7b. The -1.5% strain decreases the instability of the Ni_14_Co_2_Mn_13_Sn_3_ martensitic phase. Then, the instability of the martensitic phase decreases, and the stability of austenitic phases show a few changes. In addition, austenite is a peak at E_F_, while martensite is a pseudopotential valley. It shows that the stability of austenite is lower than that of martensite, which leads to the MPT according to the Jahn teller splitting effect. The results show that strain can tune the *T*_M_ of Ni_14_Co_2_Mn_13_Sn_3_ FSMAs. 

For the magnetic properties of Ni_14_Co_2_Mn_13_Sn_3_ austenitic phases, as can be seen in Figure 8b, the PDOS of Mn_Mn_ and Mn_Sn_ are similar and mostly up-spin states under the E_F_. With applying strain, for Figure 8a,c, the PDOS of Mn_Mn_ and Mn_Sn_ has almost no change. This is indicated that the most stable magnetic configuration of austenitic phases is always FM, and this configuration is unaffected by strain. For martensite, in Figure 9b, the Mn_Mn_ is in up-states while the Mn_Sn_ is in down-states. With applying strain, the distribution of the PDOS of Mn_Mn_ and Mn_Sn_ is still different, the Mn_Sn_ is down-state, but the Mn_Mn_ is up-state, as shown in Figure 9a,c. The most stable magnetic configuration of martensite phases is AFM. In addition, the difference in the distribution of austenite and martensite also explains the existence of large Δ*M*. In conclusion, the strain cannot change the magnetic properties of Ni_14_Co_2_Mn_13_Sn_3_ FSMAs. The Ni_14_Co_2_Mn_13_Sn_3_ FSMAs meet the condition of the stable MFIMT. 

## 4. Conclusions

To summarize, to achieve the tunable broad working temperature region of Ni-Mn-Sn alloys, we have systematically studied the influence of strain on the structures, MPT, and magnetic properties Ni_16−*x*_Co*_x_*Mn_13_Sn_3_ (*x* = 0, 1, 2) by first-principles calculation. The value of Δ*E*_A-M_ increases with the strain from 1.5% to −1.5%, bringing about *T*_M_ enhancement. According to the results, the strain method can reveal the ability of the tunable wide working temperature range in Ni_14_Co_2_Mn_13_Sn_3_ FSMAs. Particularly, with a slight strain (0~1.5%), Ni_14_Co_2_Mn_13_Sn_3_ with a large working temperature region of ~160 K and the working temperature (168–330 K) of Ni_14_Co_2_Mn_13_Sn_3_ satisfy the application from low-temperature to high-temperature. This work predicts an adjusted broad working temperature region of Ni-Mn-Sn alloys, which shows a great application range of Ni-Mn-Sn FSMAs. Therefore, the strain method provides the reference for designing the tunable wide working temperature FSMAs and makes it possible for the wide application of FSMAs. 

## Figures and Tables

**Table 1:** Equilibrium lattice parameters, total spin moments and magnetic state of the cubic austenite (Cub.) and tetragonal non-modulated martensite (Tet.) for Ni_16−*x*_Co*_x_*Mn_13_Sn_3_ (*x* = 0, 1, 2) alloys with strain (−1.5~1.5%).

Alloys	Strain %	Phase	*a* Å	*c* Å	*M*_t_ μB	|Δ*M*| μB	Magnetic State FM/AFM
Ni_16_Mn_13_Sn_3_	−1.5	Cub.	5.85	5.94	1.36	0.06	AFM
		Tet.	5.30	7.26	1.42		AFM
	−1.0	Cub.	5.88	5.94	1.37	0.05	AFM
		Tet.	5.33	7.26	1.42		AFM
	−0.5	Cub.	5.91	5.94	1.38	0.04	AFM
		Tet.	5.35	7.26	1.42		AFM
	0	Cub.	5.94	5.94	1.39	0.04	AFM
		Tet.	5.38	7.26	1.43		AFM
	0.5	Cub.	5.97	5.94	1.40	0.03	AFM
		Tet.	5.41	7.26	1.43		AFM
	1.0	Cub.	6.00	5.94	1.41	0.02	AFM
		Tet.	5.43	7.26	1.43		AFM
	1.5	Cub.	6.03	5.94	1.42	0.01	AFM
		Tet.	5.46	7.26	1.43		AFM
Ni_15_CoMn_13_Sn_3_	−1.5	Cub.	5.83	5.92	1.42	0.02	AFM
		Tet.	5.35	7.06	1.44		AFM
	−1.0	Cub.	5.86	5.92	1.44	0.01	AFM
		Tet.	5.37	7.06	1.45		AFM
	−0.5	Cub.	5.89	5.92	1.44	0.01	AFM
		Tet.	5.40	7.06	1.45		AFM
	0	Cub.	5.92	5.92	1.46	0	AFM
		Tet.	5.43	7.06	1.46		AFM
	0.5	Cub.	5.95	5.92	1.47	0	AFM
		Tet.	5.46	7.06	1.47		AFM
	1.0	Cub.	5.98	5.92	1.49	0.01	AFM
		Tet.	5.48	7.06	1.48		AFM
	1.5	Cub.	6.01	5.92	1.50	0.01	AFM
		Tet.	5.51	7.06	1.49		AFM
Ni_14_Co_2_Mn_13_Sn_3_	−1.5	Cub.	5.84	5.93	6.98	5.48	FM
		Tet.	5.32	7.15	1.50		AFM
	−1.0	Cub.	5.87	5.93	7.00	5.50	FM
		Tet.	5.35	7.15	1.50		AFM
	−0.5	Cub.	5.90	5.93	7.03	5.51	FM
		Tet.	5.37	7.15	1.52		AFM
	0	Cub.	5.93	5.93	7.06	5.54	FM
		Tet.	5.40	7.15	1.52		AFM
	0.5	Cub.	5.96	5.93	7.08	5.56	FM
		Tet.	5.43	7.15	1.52		AFM
	1.0	Cub.	5.99	5.93	7.10	5.58	FM
		Tet.	5.45	7.15	1.52		AFM
	1.5	Cub.	6.02	5.93	7.13	5.58	FM
		Tet.	5.48	7.15	1.55		AFM

**Table 2:** Calculated energy difference Δ*E*_A-M_ in meV/atom between the austenite and martensite phases, Δ*E*_P-F_ in meV/atom between the paramagnetic and ferromagnetic state, martensite transition temperature *T*_M_ and Curie temperature *T*_C_ in Ni_16−*x*_Co*_x_*Mn_13_Sn_3_ (*x* = 0, 1, 2) alloys with strain (−1.5~1.5%).

Alloys	Strain %	Δ*E*_A-M_ (meV/atom)	Δ*E*_P-F_ (meV/atom)	*T*_M_ (K)	*T*_C_ (K)
Ni_16_Mn_13_Sn_3_	−1.5	31.7	385.7	405	397
	−1.0	29.6	387.8	378	399
	−0.5	27.6	390.0	353	401
	0	24.6	393.3	314	405
	0.5	22.3	395.2	285	407
	1.0	19.4	397.6	248	409
	1.5	18.5	400.7	236	412
Ni_15_CoMn_13_Sn_3_	−1.5	30.8	392.6	394	404
	−1.0	28.1	395.9	359	407
	−0.5	26.8	397.7	343	409
	0	23.4	398.2	299	410
	0.5	21.6	403.4	276	415
	1.0	18.7	407.3	239	419
	1.5	17.8	410.4	227	422
Ni_14_Co_2_Mn_13_Sn_3_	−1.5	25.8	399.6	330	411
	−1.0	22.9	402.1	293	414
	−0.5	19.0	404.8	243	416
	0	13.2	406.9	168	419
	0.5	12.7	410.7	162	423
	1.0	9.8	413.8	125	426
	1.5	7.2	415.7	92	428

## Data Availability

The data that support the findings of this study are available from the corresponding author upon reasonable request.
